# Editorial: Diagnosis and treatment of sarcoidosis

**DOI:** 10.3389/fmed.2023.1227259

**Published:** 2023-06-13

**Authors:** Ying Zhou, Florence Jeny, Robert P. Baughman

**Affiliations:** ^1^Department of Pulmonary and Critical Care Medicine, Shanghai Pulmonary Hospital of Tongji University, Shanghai, China; ^2^Department of Pulmonology, Avicenne University Hospital, INSERM UMR 1272, Université Sorbonne Paris-Nord, Paris, France; ^3^Department of Medicine, University of Cincinnati Medical Center, Cincinnati, OH, United States

**Keywords:** sarcoidosis, diagnosis, treatment, genetic factors, clinical phenotypes

Sarcoidosis is a multisystem disease characterized by non-caseating epithelioid cell granulomas. The diagnosis of sarcoidosis is based on compatible clinical presentation, non-necrotizing granulomatous inflammation in one or more tissue samples, and the exclusion of alternative causes of granulomatous disease (1). With the development of new technology, biomarkers, CT pattern scores, and PET-CT have been applied for improved diagnosis and assessment of organ involvement. Some patients with pulmonary sarcoidosis do not require systemic treatment, given that spontaneous remission occurs at a certain rate. In the treatment of sarcoidosis, an effective individualized plan must be formulated according to the clinical manifestations, the involved organs, the severity of their involvement, and underlying conditions. The most common indication for treatment of pulmonary disease is the development of respiratory symptoms, followed by extra-pulmonary involvement of the cardiovascular, cutaneous, and nervous systems. At present, drugs for sarcoidosis include corticosteroids as first-line therapy, cytotoxic drugs as second-line treatment, and anti-tumor necrosis factor (TNF) biologics as third-line agents (2). Novel therapeutic agents such as repository corticotropin injection and rituximab have been studied for sarcoidosis and some positive results have been obtained. However, additional research efforts need to be undertaken to develop improved tools for the diagnosis and treatment of sarcoidosis. In this Research Topic, we aim to provide an overview of recent progress in the diagnosis and treatment of sarcoidosis and to present innovative solutions to existing challenges.

This Research Topic consists of one case report, three reviews, and six original research articles focusing on advances in diagnosis and treatment, together with findings in genetics and transcriptomics that might be helpful in the early diagnosis of sarcoidosis.

Multiple factors, such as environmental and genetic predisposition, have been implicated in the pathogenesis of sarcoidosis. The search for gene candidates in sarcoidosis can be conducted on the basis of gene expression data and protein profile data from genomic, transcriptomic, or proteomic studies. Xiong et al. identify sex-dependent genetic variations in two clinical sarcoidosis phenotypes, namely Löfgren's syndrome (LS) and non-Löfgren's syndrome (non-LS), in multiethnic cohorts from Sweden, Germany, and the US. The authors confirm that differences in genetic findings between the sex groups in LS are explicitly located in the extended Major Histocompatibility Complex, while genetic differences between the sex groups in non-LS are primarily located in the MHC class II subregion and *ANXA11*. Transcriptome-wide expression studies have been conducted to reveal the mechanisms of sarcoidosis. Jiang et al. performed a systematic database search of the Gene Expression Omnibus and utilized transcriptomic data from blood and sarcoidosis-affected tissues in a meta-analysis to identify a cross-tissue, cross-platform signature. They identify 29 robustly sarcoidosis-associated genes, including the top genes *LINC01278, GBP5*, and *PSMB9*. They report that pathway enrichment analysis revealed activation of IFN-γ, IL-1 and IL-18, autophagy, and viral infection response. This study provides a cross-tissue meta-analysis for expression profiles and identifies a potential non-invasive diagnostic classifier for sarcoidosis. Similar efforts have been made by Duo et al., who report on a study in which transcriptomes from eleven independent sarcoidosis cohorts comprising 313 patients and 400 healthy controls were analyzed and machine learning was employed to fit a diagnostic model. A ten-gene sarcoidosis diagnosis signature consisting of *GBP1, LEF1, IFIT3, LRRN3, IFI44, LHFPL2, RTP4, CD27, EPHX2*, and *CXCL10* was constructed in the training cohorts; this signature performed well in the four independent cohorts and has been further validated in seven independent publicly available gene expression datasets. Transcriptional signatures, developed through bioinformatics analysis, could improve the accuracy of early diagnosis of sarcoidosis.

Cardiac sarcoidosis (CS) remains diagnostically challenging as the sensitivity and specificity of the diagnostic modalities are limited. Strambu reviews current knowledge of the diagnosis and decision to treat cardiac sarcoidosis, and illustrates the information with a case presentation (Strambu). The most challenging issue is the suspicion of CS with initial cardiac manifestations in a patient with no previous diagnosis of sarcoidosis, and diagnosis can be delayed. Another difficulty in diagnosis is the absence of a gold standard for diagnosis, because biopsy of the endomyocardial tissue has low sensitivity given the risk level of the procedure. The increasing use of newer imaging modalities such as cardiac magnetic resonance (CMR) and positron emission tomography (PET) may provide valuable information for accurate diagnosis and assessment in CS patients. Brazile et al. review a set of medical records for diagnostic features of CS, including late gadolinium enhancement (LGE) patterns, increased signal on T2-weighted imaging, and non-caseating granulomas. They confirm that CMR is an important tool in the non-invasive diagnosis of CS, and the presence of LGE on CMR in a pattern consistent with CS has been shown to be a predictor of mortality.

Three manuscripts published in this Research Topic relate to rare clinical features associated with or mimicking sarcoidosis. Prevel et al. describe the clinical and biological presentation, treatments, and outcomes of *cryptococcus* spp. infection of central nervous system (CINS) in patients with and without sarcoidosis. They found that 31% (5/16) of CINS patients had associated sarcoidosis. CINS symptoms, biological and CSF features, and treatments were similar for CINS patients with and without sarcoidosis, except regarding CD4 cell percentage and CD4/CD8 ratio, which were higher in blood from those with sarcoidosis. Ji et al. present a case report of pleural sarcoidosis with pleural nodules and effusion, along with a review of the literature on this rare manifestation. Another study describes the natural course and prognostic value of cancer-associated sarcoid-like reaction (SLR) (Huh et al.). During follow-up, progression of cancer-related SLR to overt sarcoidosis was not observed. Development of SLR was also not associated with overall survival or disease-free survival in patients with non-small cell lung cancer.

Two reviews of differential diagnosis and treatment approaches are presented in our Research Topic. In the review by Valeyre et al. the authors establish optimal differential diagnosis strategies tailored to each of several situations in order to help non-sarcoidosis experts. They emphasize that alternative diagnoses must be ruled out before a diagnosis of sarcoidosis can be made. First, epidemiological factors must be clarified. Subsequently, a detailed medical history and physical examination are also crucial. Chest CT is also helpful to characterize the findings as typical or atypical for sarcoidosis. Ultimately, the Sarcoidosis Diagnostic Score (Clinical and Biopsy) can be very helpful to assess sarcoidosis diagnosis before and after evidence of granuloma. Recent therapeutic drug trials and treatment approaches are reviewed by Obi et al.. Up to now, only two medications (prednisone and repository corticotropin injection) have been approved in the treatment of sarcoidosis by the United States Food and Drug Administration. This past decade has seen a renewed interest in developing new drugs and exploring novel therapeutic pathways for the treatment of sarcoidosis. The next challenge will lie in funding and in evaluating the effectiveness and safety of treatments.

For accurate diagnosis and effective treatment, further efforts should be focused on early detection, new diagnostic technologies, and multicenter clinical trials of new drugs. Moreover, the identification of new biomarkers is warranted for differential diagnosis and for individualized treatment. This will provide a powerful decision-making tool for doctors that can be used to better manage sarcoidosis patients.

## Author contributions

The draft was written by YZ and edited by FJ and RB. All authors reviewed the final document.
